# Tumor-Stroma Crosstalk Enhances REG3A Expressions that Drive the Progression of Hepatocellular Carcinoma

**DOI:** 10.3390/ijms21020472

**Published:** 2020-01-11

**Authors:** Yuri Cho, Min Ji Park, Koeun Kim, Jae-Young Park, Jihye Kim, Wonjin Kim, Jung-Hwan Yoon

**Affiliations:** 1 Department of Internal Medicine, CHA Gangnam Medical Center, CHA University School of Medicine, Seoul 06135, Korea; jewelbox6@hanmail.net (M.J.P.); klk0915@naver.com (K.K.); hyehye08@gmail.com (J.K.); virgo914@cha.ac.kr (W.K.); 2 Department of Internal Medicine and Liver Research Institute, College of Medicine, Seoul National University, Seoul 03080, Korea; yoonjh@snu.ac.kr; 3 Department of Orthopaedic Surgery, KyungHee University Medical Center, Seoul 02447, Korea; neoxcv@gmail.com

**Keywords:** hepatocellular carcinoma, crosstalk, REG3A, stroma, hepatic stellate cell

## Abstract

Abstract: Background: Crosstalk between tumors and their microenvironment plays a crucial role in the progression of hepatocellular carcinoma (HCC). However, there is little existing information about the key signaling molecule that modulates tumor-stroma crosstalk. Methods: Complementary DNA (cDNA) microarray analysis was performed to identify the key molecule in tumor-stroma crosstalk. Subcutaneous xenograft in vivo murine model, immunoblotting, immunofluorescence, and real-time polymerase chain reaction using HCC cells and tissues were performed. Results: The key molecule, regenerating gene protein-3A (REG3A), was most significantly enhanced when coculturing HCC cells and activated human hepatic stellate cells (HSCs) (+8.2 log) compared with monoculturing HCC cells using cDNA microarray analysis. Downregulation of REG3A using small interfering RNA significantly decreased the proliferation of HSC-cocultured HCC cells in vitro and in vivo, and enhanced deoxycholic acid-induced HCC cell apoptosis. Crosstalk-induced REG3A upregulation was modulated by platelet-derived growth factor ββ (PDGF-ββ) in p42/44-dependent manner. REG3A mRNA levels in human HCC tissues were upregulated 1.8-fold compared with non-tumor tissues and positively correlated with PDGF-ββ levels. Conclusions: REG3A/p42/44 pathway/PDGF-ββ signaling plays a significant role in hepatocarcinogenesis via tumor-stroma crosstalk. Targeting REG3A is a potential novel therapeutic target for the management of HCCs by inhibiting crosstalk between HCC cells and HSCs.

## 1. Introduction

Hepatocellular carcinoma (HCC) is one of the most fatal cancers in humans with increasing incidence worldwide [1]. Despite the significant improvement in HCC management in the last decades [2], systemic target therapy, such as sorafenib, lenvatinib, or regorafenib, is the only therapy which has been demonstrated effective against advanced HCC with limited efficacy in killing residual tumors [3]. These small molecule inhibitors have limited use; only 30% of patients have the mutations targeted by these therapeutics [4]. Several attempts, largely unsuccessful, have been made to develop new drugs for HCC, indicating the presence of unknown pathogenesis of HCC [5,6,7].

The interaction between tumor cells and their microenvironment was shown to fundamentally affect cancer progression by triggering the proliferation and invasion of tumor cells. In the majority of tumors, the aberrant networks of growth factors, cytokines, and chemokines including their cognate receptors, are critically involved in cancer progression [8,9]. Crosstalk between tumor cells and their microenvironment also plays a crucial role in the progression of HCC. The majority of HCC patients have a history of chronic liver disease, and the presence of liver cirrhosis is the main risk factor for the development of HCC. Activated hepatic stellate cells (HSCs) are the effector cells of hepatic fibrosis [10]. Upon hepatic injury, HSCs transform to an activated, myofibroblast-like phenotype that is responsible for the excessive hepatic matrix deposition in chronically damaged livers [11,12]. Consequently, HSCs proliferate and migrate towards the area of ongoing tissue remodeling and secrete extracellular matrix proteins [13]. HSCs also infiltrate the HCC stroma where they might play a critical role in tumor progression as well as angiogenesis [14,15]. Increased understanding of how stroma interacts with tumor cells and the signaling pathways involved could help identify new therapeutic targets.

We recently reported that HSC-derived platelet-derived growth factor ββ (PDGF-ββ) stimulates the proliferation of HCC cells by activating the phosphoinositide 3-kinase (PI3K)/Akt pathway, while the inhibition of PDGF-ββ or PI3K/Akt pathways enhances apoptotic cell death, especially under hypoxic conditions [16]. In the present study, the key molecule that modulates crosstalk between HCC cells and their microenvironment was investigated using complementary DNA (cDNA) microarray.

## 2. Results

### 2.1. Coculturing HCC Cells and HSCs Enhanced REG3A Expression

**Figure 1:**
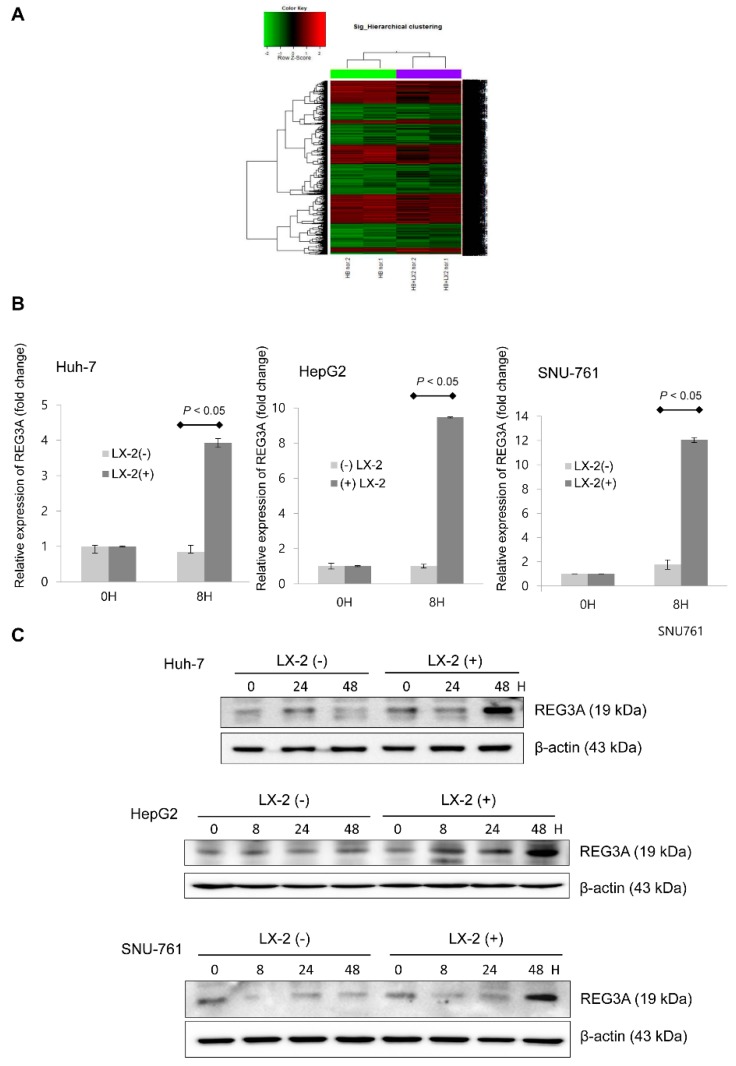
Coculture of hepatocellular carcinoma (HCC) cells and hepatic stellate cells (HSCs) enhanced the expression of regenerating gene protein-3A (REG3A). (**A**) Complementary DNA (cDNA) microarray results (volume plot of expression level, hierarchical clustering analysis). (**B**) REG3A mRNA in HCC cells was significantly enhanced when coculturing with HSCs compared with monoculturing. REG3A mRNA was quantified using real-time polymerase chain reaction (qPCR) and normalized to glyceraldehyde-3-phosphate dehydrogenase (GAPDH) expression levels. The experiment was repeated three times. The data are expressed as mean ± SD. (**C**) The protein expression of REG3A in HCC cells was significantly enhanced when coculturing with HSCs compared with monoculturing, especially at 48 h. The experiment was repeated three times.

Using cDNA microarray analysis, the key molecule, REG3A, was significantly enhanced when coculturing Huh-7 cells and HSCs (+ 8.2 log) compared with monoculturing Huh-7 cells (Figure 1A). Coculturing HCC cells (Huh-7, HepG2, and SNU-761) with HSCs enhanced the mRNA (Figure 1B) and protein expressions (Figure 1C) of REG3A compared with monoculturing HCC cells.

### 2.2. Modulation of REG3A in Cocultured HCC Cells Showed Anti-Tumor Effects In Vitro and In Vivo

**Figure 2:**
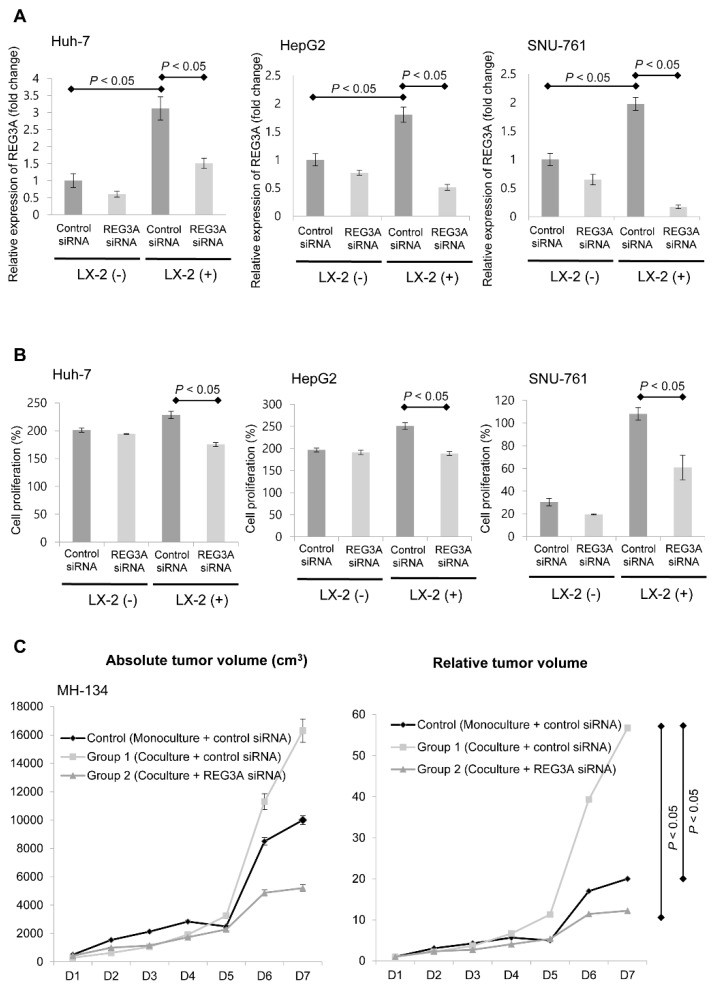
The effects of REG3A on proliferation of HCC cells cocultured with HSCs. (**A**) REG3A small interfering RNA (siRNA) transfection significantly suppressed REG3A mRNA expression as compared to control siRNA in HCC cells (*p* < 0.05). Data are expressed as mean ± SD of percent changes of optical densities. The experiment was repeated three times. (**B**) When HCC cells were transfected with REG3A siRNA, the proliferation of HCC cells was significantly decreased compared with control siRNA transfection based on the 3-(4,5-dimethylthiazol-2-yl)-2,5-diphenyl tetrazolium bromide (MTT) assay results (*p* < 0.05). Data are expressed as mean ± SD of percent changes of optical densities. The experiment was repeated three times. (**C**) Coculturing MH-134 cells with HSCs (Group 1) enhanced the proliferation of HCC cells compared with monoculturing MH-134 cells (Control) (*p* < 0.05). REG3A siRNA attenuated in vivo HCC cell proliferation (Group 2) compared with control siRNA transfection (Group 1) (*p* < 0.05). The data are expressed as mean ± SD.

First, we examined the effective REG3A siRNA transfection with RT-PCR. As shown in Figure 2A, REG3A siRNA transfection significantly suppressed REG3A mRNA expression as compared to control siRNA, on HCC cells (*p* < 0.05).

Then, we performed MTT assay to evaluate whether REG3A modulates HCC cell proliferation. The potential antiproliferative effects of downregulated REG3A was investigated using siRNA in vitro when HCC cells were cocultured with HSCs. Downregulation of REG3A caused by siRNA significantly decreased the proliferation of tumor cells in vitro (Figure 2B; both *p* < 0.05).

Antitumor effects of the REG3A siRNA were examined using an in vivo xenograft model. The growth of liver tumor was significantly enhanced in Group 1 (control siRNA transfected MH134 cell + LX-2 coculturing) compared with the control group (control siRNA transfected MH134 cell), especially at day 7 (D7; *p* < 0.05). Tumor growth induced when coculturing HCC cells and HSCs was also significantly inhibited following REG3A siRNA transfection in Group 2 (REG3A siRNA transfected MH134 cell + LX-2 coculturing) compared with Group 1, especially at D7 (Figure 2C; *p* < 0.05).

### 2.3. Downregulation of REG3A Decreased Bile Acid-Induced HCC Cell Apoptosis

**Figure 3:**
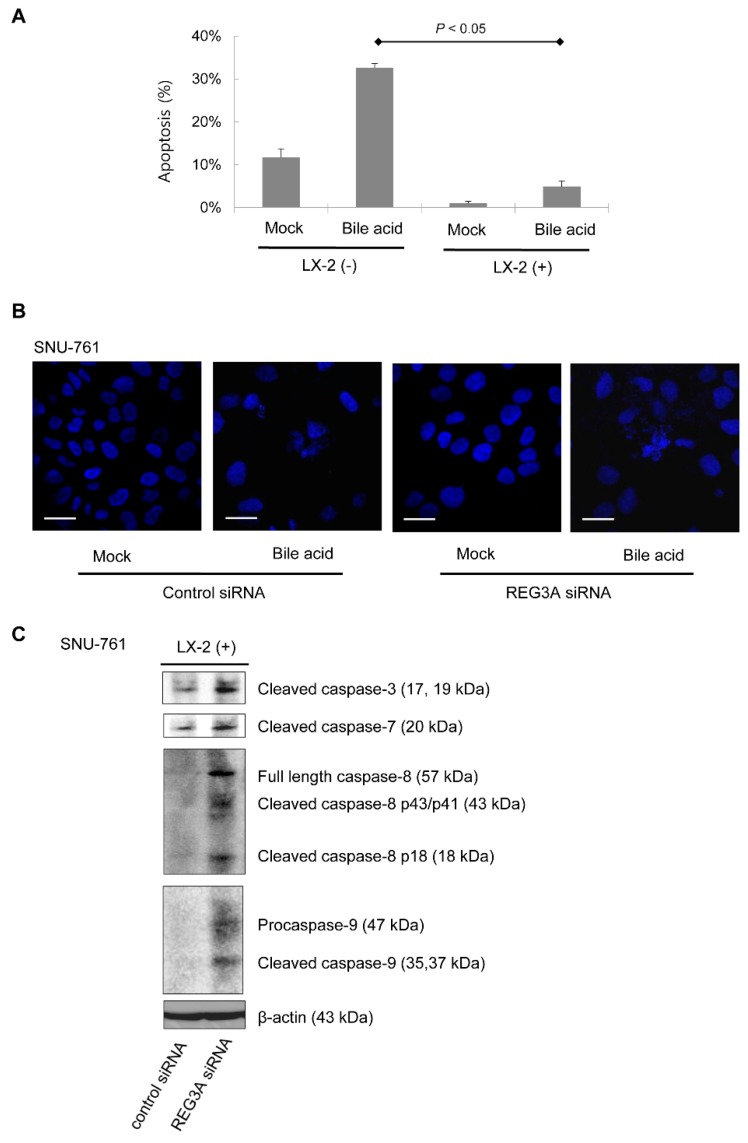
The effects of REG3A on bile acid-induced apoptosis of HCC cells cocultured with HSCs. (**A**) SNU-761 cells were monocultured or cocultured with LX-2. After 24 h, cells were treated with deoxycholate (300 μM) for 2 h. Apoptosis was assessed using 40,6-diamidino-2-phenylindole dihydrochloride (DAPI) staining. Data are expressed as mean ± SD from three different experiments (*p* < 0.05, vs. mono-culturing without LX-2). The experiment was repeated three times. (**B**) On fluorescence microscopy, SNU-761 cells cocultured with LX-2 cells were significantly more resistant to bile acid (deoxycholic acid 300 μM)-induced SNU-761 cell apoptosis compared with monocultured cells. Scale bars, 50 μm. (**C**) Immunoblot analyses of caspase 3, 7, 8, and 9 were performed in SNU-761 cells cocultured with LX-2 cells. The experiment was repeated three times.

SNU-761 cells cocultured with LX-2 cells were significantly more resistant to bile acid (deoxycholic acid 300 μM)-induced SNU-761 cell apoptosis compared with monocultured cells (Figure 3A). Next, the effects of REG3A on cellular apoptosis under coculturing conditions were evaluated. Immunofluorescence results also showed that bile acid-induced SNU-761 cell apoptosis was inhibited when REG3A was downregulated in HSC-cocultured SNU-761 cells (Figure 3B). In immunoblot analyses, the protein expressions of caspase 3, 7, 8, and 9 were upregulated in SNU-761 cells cocultured with LX-2 cells (Figure 3C).

### 2.4. The Antitumor Mechanism of REG3A in HCC Cells Cocultured with HSCs: p42/44 Pathway

**Figure 4:**
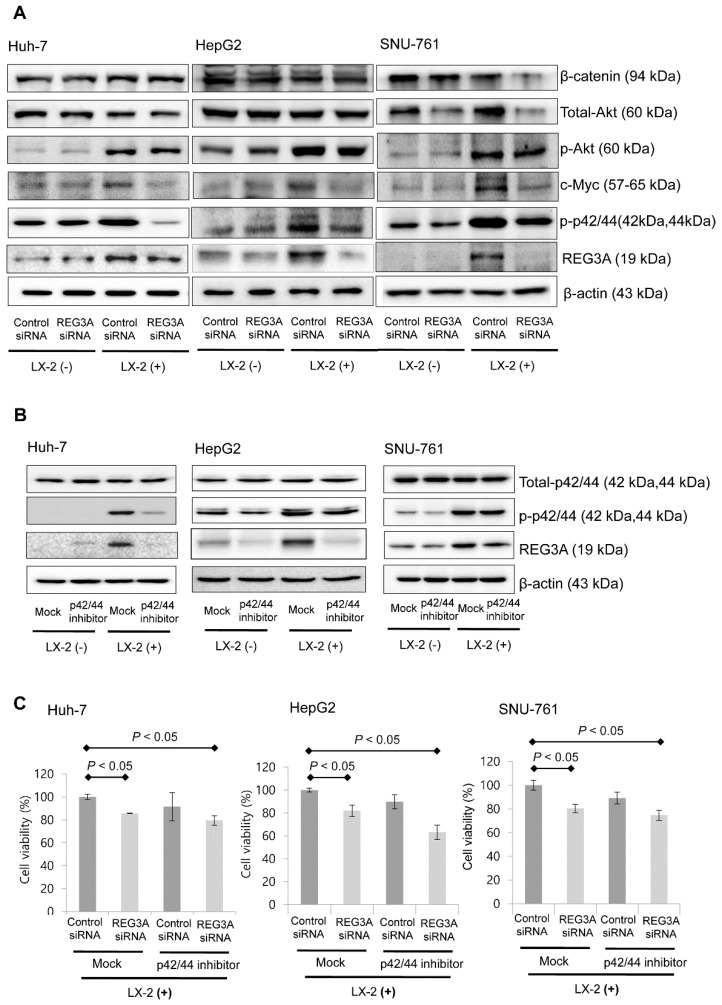
The p42/44 pathway-dependent REG3A activation modulates the expression of platelet-derived growth factor ββ (PDGF-ββ). (**A**) Immunoblot analyses of β-catenin, p-Akt, total Akt, c-myc, phosphorylated p42/44, total p42/44, and REG3A were performed in cocultured or monococultured HCC cells with REG3A siRNA or control siRNA transfection. The experiment was repeated three times. (**B**) P42/44 inhibitor (PD98059, 10 μM) significantly decreased the protein expression of REG3A in cocultured HCC cells. The experiment was repeated three times. (**C**) Downregulating REG3A significantly suppressed HCC cell proliferation based on the MTT assay results (*p* < 0.05). The experiment was repeated three times. The data are expressed as mean ± SD.

The immunoblot assay results showed downregulation of REG3A caused by siRNA transfection decreased the expression of phosphorylated p42/44 (p’-p42/44), especially when HCC cells were cocultured with HSCs (Figure 4A). When HCC cells were treated with p42/44 inhibitor (PD98059, 10 μM), the protein expression of REG3A significantly decreased compared with control (Figure 4B). Results of densitometric analysis are presented as the relative ratio of REG3A to β-actin (Figure S1).

Downregulation of REG3A also significantly suppressed HCC cell proliferation based on MTT assay results (*p* < 0.05). Single p42/44 inhibitor treatment did not suppress HCC cell proliferation significantly. However, after REG3A-siRNA transfection, a significant antiproliferative effect was observed (Figure 4C).

### 2.5. Crosstalk-Induced REG3A Upregulation was Modulated by PDGF-ββ

**Figure 5:**
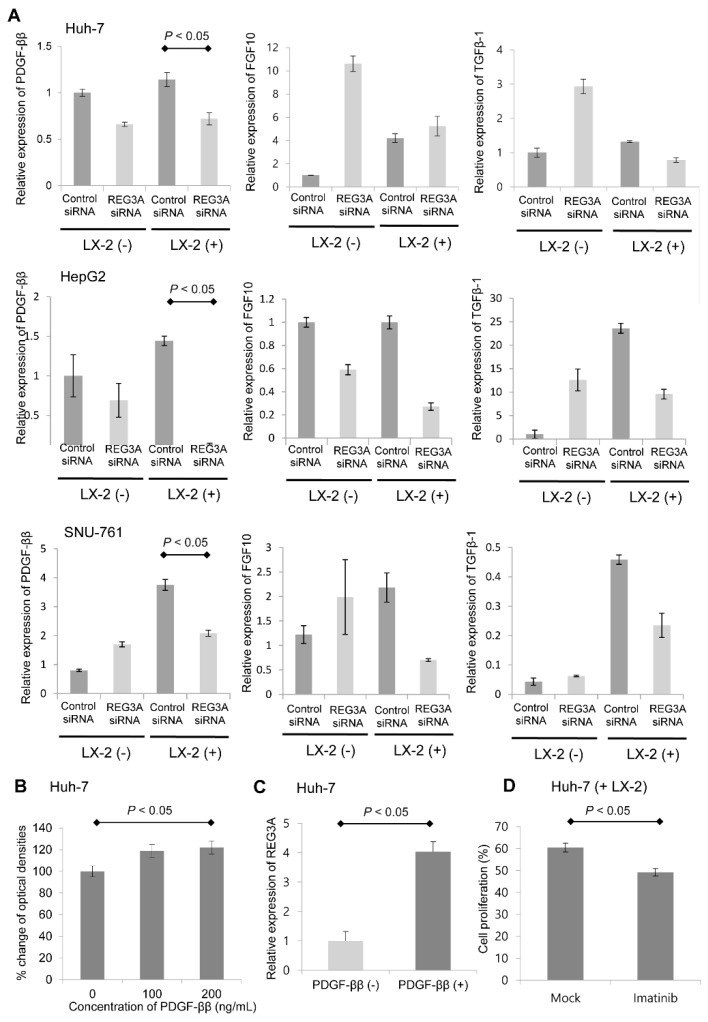
REG3A enhanced HCC proliferation by reciprocally influencing PDGF-ββ. (**A**) REG3A siRNA significantly decreased the mRNA expression of PDGF-ββ in cocultured HCC cells (*p* < 0.05). The experiment was repeated three times. The data are expressed as mean ± SD. (**B**) Exogenous PDGF-ββ (200 ng/mL) enhanced the proliferation of HCC cells based on the MTT assay results (*p* < 0.05). The experiment was repeated three times. The data are expressed as mean ± SD. (**C**) Exogenous PDGF-ββ enhanced the expression of REG3A in cocultured HCC cells. The experiment was repeated three times. The data are expressed as mean ± SD. (**D**) Huh-7 cells were treated with PDGF receptor inhibitor (imatinib, 10 μM) under coculturing with LX-2. After 24 h, an MTT assay revealed that imatinib significantly suppressed the proliferation of Huh-7 cells (*p* < 0.05). Data are expressed as mean ± SD of percent changes of triplicate optical densities compared to that of control.

The mRNA expression of HSC-derived, HCC-relevant growth factors such as TGF-β1, FGF10, and PDGF-ββ was evaluated in LX-2 cells. Among the growth factors tested, PDGF-ββ exhibited the most significant fold-change in mRNA expression levels. Notably, crosstalk-induced PDGF-ββ upregulation was modulated by REG3A siRNA transfection (Figure 5A; *p* < 0.05). Next, whether REG3A enhanced HCC cell proliferation by reciprocally influencing PDGF-ββ was investigated. As shown in Figure 5B, exogenous PDGF-ββ (200 ng per mL) significantly enhanced the proliferation of HCC cells. Exogenous PDGF-ββ also upregulated the mRNA expression of REG3A in cocultured HCC cells, leading to HCC cell proliferation (Figure 5C). Huh-7 cells were treated with PDGF receptor inhibitor (imatinib, 10 μM) under coculturing with LX-2. After 24 h, an MTT assay was performed. Imatinib significantly suppressed the proliferation of Huh-7 cells under coculturing with LX-2 (Figure 5D; *p* < 0.05).

### 2.6. Upregulated mRNA Expression of REG3A was Correlated with the Expression of PDGF-ββ in Human HCC Tissues

**Figure 6:**
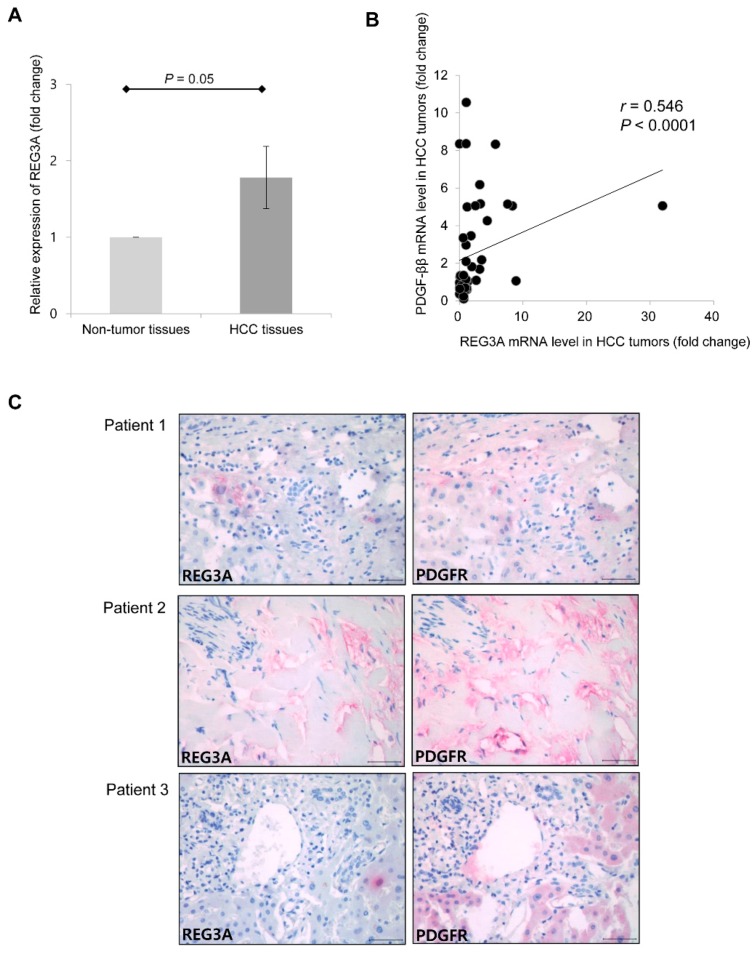
The mRNA level of REG3A was positively correlated with PDGF-ββ in human HCC tissue. (**A**) The mean mRNA expression of REG3A was upregulated 1.8-fold in HCC tissues compared with non-tumor tissues (*n* = 88). The data are expressed as mean ± SD. (**B**) The mRNA expression of REG3A was positively correlated with PDGF-ββ (Pearson’s coefficient = 0.546; *p* < 0.001). (**C**) Expression of REG3A and PDGF receptor (PDGFR) in three human HCC tissues were detected by immunohistochemistry (400 × magnification). Scale bars, 50 µm.

For gene expression analyses, 88 surgically resected frozen HCC tumor and 88 paired non-tumor liver tissue samples were evaluated. The majority of patients (*n* = 71, 80.7%) were stage I according to the American Joint Commission on Cancer 8th edition HCC staging system. Eleven patients (12.5%) and six patients (6.8%) were stage II and stage III, respectively. No patient had major vascular invasion or lymph node/distant metastasis. The expression of REG3A was further determined in resected HCC and adjacent non-tumor tissues using qRT-PCR. The mean mRNA expression of REG3A was upregulated 1.8-fold in HCC tissues compared with non-tumor tissues (Figure 6A; *p* = 0.05). Among 88 HCC tumor tissues, the REG3A mRNA expression was upregulated in 46 samples (52.3%) as compared to non-tumor tissues, and positively correlated with PDGF-ββ (Figure 6B; Pearson’s coefficient = 0.546; *p* < 0.001). Immunohistochemical study using three human HCC tissues also revealed that the expression of REG3A was accompanied by an increased expression of PDGF receptor (PDGFR) (Figure 6C).

## 3. Discussion

**Figure 7:**
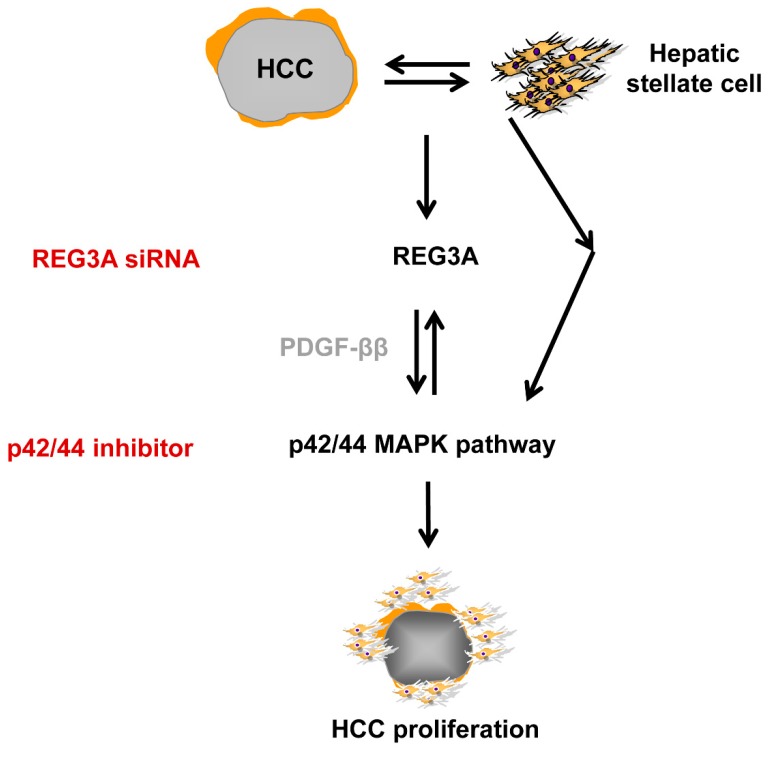
Graphical abstract. REG3A/p42/44 pathway/PDGF-ββ signaling plays a significant role in hepatocarcinogenesis via tumor-stroma crosstalk.

REG3A is a key molecule modulated by crosstalk between HCC cells and HSCs. The interaction between tumor cells and their microenvironment fundamentally affects cancer development by triggering cell proliferation and survival for subsequent spreading and colonization [17]. HSCs are activated in response to PDGF-ββ or TGF-β [18]. In the present study, REG3A was shown to modulate the expression of PDGF-ββ in HCC cells crosstalking with HSCs in the p42/44-dependent pathway (Figure 7).

We previously established hypoxic HSC-derived PDGF-ββ stimulates the proliferation of HCC cells by activating the PI3K/Akt pathway, while the inhibition of PDGF-ββ or PI3K/Akt pathway enhances apoptotic cell death, indicating targeting tumor-stroma crosstalk might be a novel therapy for the management of human HCC [14]. The results from the current study support this hypothesis and indicate REG3A might be the key targeting molecule activated by crosstalk between HCC cells and tumor stroma.

REG3A is a 19 kD secretory pancreas protein with pro-growth function. REG3A is a member of the Reg protein family and other members of the Reg protein family are associated with human gastric cancers. In previous studies, the altered expression of REG3A in gastrointestinal cancers has been investigated [19,20,21]. One pilot study reported the role of REG3A in modulating pancreatic ductal adenocarcinoma which interacts with WNT/CTNNB1 and TGF-beta pathways [22]. REG3A was originally identified as a pancreatitis-associated protein (PAP) released by the acini during acute pancreatitis, and is a secreted C-type lectin protein reportedly upregulated in primary HCC, although REG3A expression was not detected in normal liver tissue [23,24]. However, current knowledge regarding the underlying mechanisms is limited and tissue specificity is an important aspect of the genetic modification of HCC susceptibility. In several functional studies, REG3A was shown possibly involved in cell recognition and adhesion as well as in the protection of cells from oxidative stress-induced apoptosis [25,26]. Cavard et al. previously reported overexpression of REG1A and REG3A in human primary liver tumor with β-catenin mutations [27]. The novel finding of this present study is that the REG3A mRNA and protein expression levels were significantly enhanced in HCC cells as a result of crosstalk between tumor and stroma. P42/44 inhibitor inhibited the expression of REG3A, indicating the silencing of the REG3A gene in HCC cell lines might be modulated by the p42/44-dependent pathway. Recently, biological and microenvironmental drivers of carcinogenesis have been reported by many researchers in gastrointestinal tumors [20,22]. Interactions between cancer cells and the abnormal bystander cells supply have been highlighted as critical factor in tumor cell proliferation and survival as well as in angiogenesis and metastasis [15,28]. The present investigation of molecular and genetic alterations in HCC by HSCs is one of the evidences of which provide useful insights into the pathogenesis of tumor cells and their surrounding microenvironment. By means of targeting immuno-microenvironment, REG3A might be a novel molecular classification of hepatocarcinogenesis [29].

We previously reported that hypoxia activates PDGF-ββ and its receptor, which was augmented by crosstalk between HCC cells and HSCs [16]. Our data showed crosstalk-induced PDGF-ββ was also modulated by REG3A. Intra- and peritumoral accumulation of PDGF-ββ arising from activated HSCs developed HCC from malignant hepatocytes. Targeting REG3A also suppressed the expression of PDGF-ββ in the present study. These results indicate pharmacological targeting of REG3A might be an effective therapy for interfering with both the malignant progression of hepatocytes and the tumor promoting consequences of hepatic stroma.

The only systemic therapies approved for advanced HCC include the multikinase inhibitors such as sorafenib, regorafenib, and lenvatinib [30,31]. A modest survival advantage was observed in HCC patients treated with sorafenib, regorafenib, or lenvatinib [32]. Recent evidence shows that long-term treatment leads to sorafenib resistance in HCC patients because tumor-driving pathways including Akt, become activated [33,34], indicating the involvement of an unknown molecular pathway in hepatocarcinogenesis. Moreover, HCCs frequently display heterogeneous growth patterns and/or cytologic features within the same tumor [35], possibly reflecting either plasticity of phenotypes or intratumor genetic heterogeneity. Molecular analysis, based on testing a small piece of tumor, might underestimate the complexity of tumor genomics. Therefore, exploring the molecular pathways involved in hepatocarcinogenesis might provide new therapeutic targets in HCC and molecular-based individualized therapies, not underestimating the tumor microenvironment and its roles in angiogenesis or immune system [36].

## 4. Materials and Methods

### 4.1. Cell Lines and Coculture

Human HCC cell lines and an HSC line were used in this study: Huh-7, HepG2, well-differentiated HCC cell lines [37]; SNU-761, a poorly differentiated HCC cell line [38]; and LX-2, an activated, human HSC line [39]. Cells were grown in Dulbecco’s Modified Eagle Medium (DMEM; Huh-7, HepG2, and LX-2) or in RPMI 1640 (SNU-761) supplemented with 10% fetal bovine serum (FBS), 100,000 U/L penicillin, and 100 mg/L streptomycin, with or without 100 nM insulin. Coculture experiments were performed in serum-free DMEM or RPMI 1640 using 1-μm pore size transwell inserts (Corning; Lowell, MA, USA), which permitted diffusion of media but prevented cell migration. HCC and LX-2 cell lines were incubated alone, or side-by-side, using transwell inserts under standard culture conditions (20% O_2_ and 5% CO_2_, at 37 °C).

### 4.2. Cell Proliferation Analysis (3-(4,5-Dimethylthiazol-2-yl)-2,5-Diphenyltetrazolium Bromide (MTT) Assay)

Using the Cell Titer 96 Aqueous One Solution cell proliferation assay (Promega, Madison, WI, USA), cell proliferation was measured based on cellular conversion of the colorimetric reagent 3-(4,5-dimethylthiazol-2-yl)-2,5-diphenyltetrazolium bromide (MTT) into soluble formazan by the dehydrogenase enzyme found in metabolically proliferating cells. Following each treatment, 20 μL of dye solution was added into each well in the 96-well plate and incubated for 2 h. Subsequently, the absorbance was recorded at a wavelength of 490 nm using an enzyme-linked immunosorbent assay (ELISA) plate reader (Molecular Devices, Sunnyvale, CA, USA).

### 4.3. Apoptosis Analysis

Chromatic condensation and nuclear fragmentation were assessed using a DNA binding dye, 40,6-diamidino-2-phenylindole dihydrochloride (DAPI), and fluorescence microscopy (Carl Zeiss; Jena, Germany).

### 4.4. cDNA Microarray Analysis

To compare relative gene expression profiles, total RNA from Huh-7 cells cocultured with HSCs or monocultured Huh-7 cells were extracted and purified. Microarray analysis was performed according to the Macrogen Rat BeadChip technical manual (Macrogen, Seoul, Korea) using the Illumina RatRef-12 Expression BeadChip (Illumina, Inc., San Diego, CA, USA). Biotinylated cRNAs were prepared from 0.55 μg quantities of total RNA using the Illumina TotalPrep RNA Amplification Kit (Ambion, Austin, TX, USA). Following fragmentation, cRNA was hybridized to the Illumina RatRef-12 Expression BeadChip (Illumina, San Diego, CA, USA ) in 0.75 μg quantities using protocols provided by the manufacturer. Arrays were scanned using the Illumina Bead Array Reader Confocal Scanner (Illumina, San Diego, CA, USA). Array data export processing and analysis were performed using Illumina BeadStudio v3.1.3 (Gene Expression Module v3.3.8).

### 4.5. Small Interfering RNA (siRNA) Transfection

Cells were seeded in a 6-well culture plate (2 × 10^5^ cells per well) in 2-mL antibiotic-free medium supplemented with 10% FBS. At 60%–80% confluence, the cells were transfected with small interfering RNA (siRNA) using the siRNA Transfection Reagent (Santa Cruz Biotechnology Inc., Santa Cruz, CA, USA) according to the manufacturer’s instructions. The cells were treated with siRNA for 6 h at 37 °C and then growth medium containing 20% FBS and antibiotics was added. After 18 h, the medium was replaced with fresh medium containing 10% FBS and antibiotics. At 24 h after the transfection, the cells were used in further experiments.

### 4.6. In Vivo Subcutaneous Xenograft Model

Briefly, control siRNA transfected-MH134 cells (5 × 10^7^ cells per mouse) were subcutaneously transplanted into the flanks of C3H mice (male, 5 weeks old) in the control group (*n* = 10). Control siRNA transfected-MH134 cells (5 × 10^7^ cells per mouse) and HSCs (5 × 10^5^ cells per mouse) were subcutaneously transplanted into the mice in Group 1 (*n* = 10). Regenerating gene protein (REG) 3A siRNA transfected-MH134 cells (5 × 10^7^ cells per mouse) and HSCs were subcutaneously transplanted into the mice in Group 2 (*n* = 10). Tumor volume was measured using a Vernier caliper and calculated as (length × (width)^2^)/2. The maximal diameter and volume for each nodule were measured daily for 10 days.

### 4.7. Immunoblot Analysis

Cells were lysed for 20 min on ice with lysis buffer and centrifuged at 14,000× *g* for 10 min at 4 °C. Samples were resolved using sodium dodecyl sulfate polyacrylamide gel electrophoresis (SDS-PAGE), transferred to nitrocellulose membranes, blotted with appropriate primary antibodies at a dilution of 1:1000, and treated with peroxidase-conjugated secondary antibodies (Biosource International, Camarillo, CA, USA). Bound antibodies were visualized using chemiluminescent substrate (ECL; Amersham, Arlington Heights, IL, USA) and exposed to Kodak X-OMAT film (Kodak, New Haven, CT, USA). Rabbit anti-regenerating gene protein-3A (REG3A) was obtained from Abcam (ab95316). Primary antibodies including rabbit anti-phospho-p42/44 mitogen-activated protein kinase (MAPK), anti-phospho-Akt, rabbit anti-c-myc, rabbit anti-caspase 8, anti-caspase 9, and anti-caspase 7 (cleaved) were purchased from Cell Signaling Technology (Danvers, MA, USA). Goat anti-actin antibody was obtained from Santa Cruz Biotechnology Inc. (Santa Cruz, CA, USA). Densitometric analyses were performed using Image J software (National Institutes of Health, Bethesda, MD, USA).

### 4.8. Real-Time Polymerase Chain Reaction (qPCR) Analysis

Total ribonucleic acids (RNAs) were extracted from Huh-7, HepG2, and SNU-761 cells using Trizol Reagent (Invitrogen, Carlsbad, CA, USA). cDNA templates were prepared using oligo(dT) random primers and Moloney Murine Leukemia Virus (MoMLV) reverse transcriptase. After the reverse transcription reaction, the cDNA template was amplified with polymerase chain reaction (PCR) using Taq polymerase (Invitrogen). REG3A was quantified with real-time PCR (qPCR; LightCycler; Roche Molecular Biochemicals, Mannheim, Germany) using SYBR green as the fluorophore (Molecular Probes, Eugene, OR, USA). Primers for REG3A were as follows: forward: 5‘-CACAGCATTTCTGAGGTGGA-3’; and reverse: 5‘-GAATGAGGTGGTCAGGTTGG-3’. Glyceraldehyde-3-phosphate dehydrogenase (GAPDH) gene expression was used as a control. The level of REG3A mRNA expression was calculated as the relative intensity of the PCR product bands compared with the GAPDH gene using the 2^−ΔΔCt^ method. The mRNA expressions of human fibroblast growth factor 10 (FGF10), transforming growth factor-β1 (TGF-β1), and PDGF-ββ were also assessed. All PCR experiments were performed three times.

### 4.9. Statistical Analyses

Statistical analyses were performed using PASW version 21.0 (SPSS Inc., Chicago, IL, USA). All experimental results were obtained from three independent experiments using cells from three separate isolations and presented as mean ± standard deviation (SD). Mann–Whitney test was used for non-parametric measures. For comparisons between groups, data were analyzed using the Mann–Whitney *U* test or one-way ANOVA. For all tests, *p* < 0.05 was considered statistically significant.

### 4.10. Ethics Statement

Ethics approval was obtained from the ethics committee of CHA University. This study was conducted in strict accordance with the recommendations from the Guide for the Care and Use of Laboratory Animals of the National Institutes of Health. In vivo study protocol was approved on 30 March2017 by the Institutional Animal Care and Use Committee (IACUC-170057) of CHA University. All in vivo surgical procedures were performed under anesthesia with 2,2,2-tribromoethanol and all efforts were made to minimize suffering.

All experiments using human tissue were approved on 13 March 2018 by the Bundang CHA Medical Center Institutional Review Board (CHAMC 2018-02-037). All human tissues were provided by Bundang CHA Biobank of Bundang CHA Medical Center. For gene expression analyses, 88 surgically resected frozen HCC and 88 non-tumor liver tissue samples were analyzed. Cases were prospectively and consecutively identified at Bundang CHA Medical Center between 2012 and 2018 (92 frozen tissue samples).

## 5. Conclusions

In summary, REG3A is a key molecule modulated by crosstalk between HCC cells and their microenvironment. The interaction between tumor and stroma triggered HCC proliferation and survival in response to PDGF-ββ. The results from the present study indicate that REG3A/p42/44 pathway/PDGF-ββ signaling plays a significant role in hepatocarcinogenesis via tumor-stroma crosstalk. Targeting REG3A is a potential novel therapeutic target for the management of human HCCs by inhibiting crosstalk between HCC cells and HSCs.

## Supplementary Materials

The following are available online at https://www.mdpi.com/1422-0067/21/2/472/s1, Figure S1: Densitometric analysis for the relative ratio of REG3A.

## Abbreviations

HCC	hepatocellular carcinoma
HSC	hepatic stellate cell
cDNA	complementary DNA
siRNA	small interfering RNA
WME	William’s medium E
MTT	3-(4,5-dimethylthiazol-2-yl)-2,5-diphenyltetrazolium bromide
ELISA	enzyme-linked immunosorbent assay
PCR	polymerase chain reaction
DMEM	Dulbecco’s Modified Eagle Medium
FBS	fetal bovine serum
GAPDH	glyceraldehyde-3-phosphate dehydrogenase
SD	standard deviation
REG3A	regenerating gene protein 3
FGF10	fibroblast growth factor 10
TGF-β1	transforming growth factor-β1
PDGF	platelet-derived growth factor
PI3K	phosphoinositide 3-kinase
MAPK	mitogen-activated protein kinase
