# Supplementary figures and images for: Tumor-Stroma Crosstalk Enhances REG3A Expressions that Drive the Progression of Hepatocellular Carcinoma
Source: Int J Mol Sci. 2020 Jan 11;21(2):472. doi: 10.3390/ijms21020472 (PMC7013972; doi:10.3390/ijms21020472)

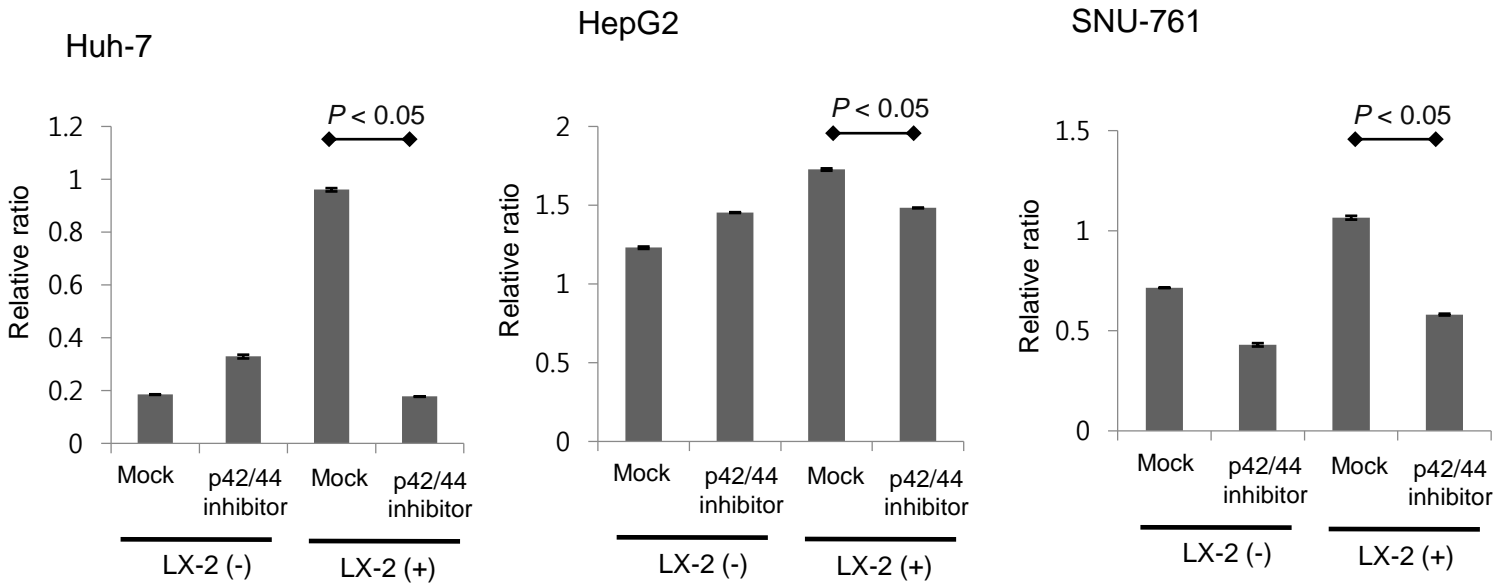

Supplement: Supplementary file 1 [file ijms-21-00472-s001.pdf]
